# Difficult Diagnoses in Hyperkinetic Disorders – A Focused Review

**DOI:** 10.3389/fneur.2012.00151

**Published:** 2012-10-29

**Authors:** Francisco Cardoso

**Affiliations:** ^1^Department of Internal Medicine, The Federal University of Minas GeraisBelo Horizonte, Minas Gerais, Brazil

**Keywords:** tremor, essential tremor, Parkinson’s disease, dystonic tremor, chorea, Sydenham’s chorea

## Abstract

Hyperkinesias are heterogeneous conditions that share the feature of production of involuntary, abnormal, excessive movements. Tremor, dystonia, and chorea are amongst the most common of these phenomena. In this focused review there is a discussion of difficult issues in hyperkinesias. The first one is the differential diagnosis between essential tremor (ET) and Parkinson’s disease (PD). They are readily distinguishable in the majority of patients but in a few subjects ET coexist with parkinsonian features whose underlying mechanism remains to be determined. The second topic of the review is dystonic tremor. Although increasingly diagnosed and reported as accounting for the majority of scans without evidence of dopaminergic deficits, its diagnostic criteria are ill-defined and differentiation from PD and ET can be challenging. In the last section, there is a discussion of the differential diagnosis of Sydenham’s chorea (SC), the most common cause of chorea in children. In a few patients, vascular disease, systemic lupus erythematosus, and primary antiphospholipid antibody syndrome can mimic SC.

## Introduction

The term “hyperkinetic disorders” encompasses a large number of heterogeneous conditions that share the feature of production of involuntary, abnormal, excessive movements. The aim of this article is to discuss problems related to the diagnosis of some of the disorders meeting this inclusion criterion. Given the wide variety of hyperkinetic disorders, the author has made a choice of topics to be tackled, which can be regarded as arbitrary but they represent issues that have been source of diagnostic controversy in the recent literature.

Tremor is defined as a movement disorder characterized by oscillatory, often rhythmic movement resulting from alternating contraction of muscles. There is consensus that it is the most common movement disorder in population-based studied, although the majority of patients attending movement disorders units have the diagnosis of parkinsonism (Fahn and Jankovic, 2007). In the field of tremor, this article will discuss the controversial relationship between essential tremor (ET) and Parkinson’s disease (PD). Abnormal postures and/or twisted movements produced by repetitive and patterned muscle contractions are the definition of dystonia (Fahn and Jankovic, 2007). In the last few years, the term “dystonic tremor” has been used to describe a condition about which there is a rather extensive body of work. One of the aims of this article is to discuss the definition and diagnosis of dystonic tremor. Chorea is a word derived from the Greek term to refer to dance. This is an apt choice of terminology since the continuous and random flow of muscle contractions that characterize chorea gives this movement disorder an appearance of dance (Cardoso et al., 2006).

## Differentiation between Essential Tremor and Parkinson’s Disease

The current definition of ET developed by the Movement Disorders Society states that patients have bilateral postural or kinetic tremor involving hands and forearms that is visible and persistent and/or tremor of the head without abnormal posturing. Exclusion criteria are other abnormal neurological signs (especially dystonia), presence of known causes of enhanced physiological tremor, evidence of psychogenic tremor, convincing evidence of sudden onset or evidence of stepwise deterioration, primary orthostatic tremor, isolated voice tremor, isolated position-specific or task-specific tremors, isolated tongue or chin tremor, and isolated leg tremor (Deuschl et al., 1998). In contrast, according to the Queen Square Brain Bank, the most commonly used set of diagnostic criteria of PD, the latter is diagnosed when the patient has bradykinesia and at least one of following cardinal signs of parkinsonism: rigidity, tremor, and postural instability, in the absence of a long list of exclusion criteria. Furthermore, there are additional supportive features, of which the most important one is response to l-DOPA (Gibb and Lees, 1998).

Strictly considering these two definitions, there is little reason to believe that there might be difficulties in distinguishing ET from PD. In fact, in most cases the differential diagnosis is readily made. As it has been described in many recent reviews, most authors lean toward the opinion that there is no relationship between the two conditions (Adler et al., 2011). This is supported by the lack of shared clinical features (e.g., olfaction is normal in ET and reduced in PD; sleep abnormalities, such as REM sleep behavioral disorder is not present in ET); genetic markers (genes associated to PD are unrelated to ET and vice-versa); pathological findings (synucleinopathy is not found in ET subjects); and therapeutic response (Adler et al., 2011; Fekete and Jankovic, 2011). Nevertheless, epidemiological data suggest that there might be a relationship between ET and PD (La Roia and Louis, 2011), although some argue that this is due to inappropriate diagnosis of the former. There are, however, few instances where the distinction is not clear-cut. The most common source of problem are patients with a longstanding history of action tremor, improvement with use of ethanol, and family history of a similar condition who later in life develop worsening of the condition with onset of rest tremor and bradykinesia (Fekete and Jankovic, 2011). Conversely, patients with PD who present with prominent postural and kinetic tremor blur the clinical distinction between PD and ET. Even those who do not believe there is a relationship between ET and PD concede that these patients point out to the existence of a link between the two conditions (Adler et al., 2011). However, although clinically these patients meet criteria of both PD and ET, post mortem studies of a few of them have failed to demonstrate that there is underlying Lewy body pathology (Louis et al., 2011). One can conclude, thus, that a few patients with long history of ET eventually develop parkinsonism but not PD. It remains, thus, to be elucidated the mechanism underlying the parkinsonian findings of these patients. In clinical practice, there are a few points that need to be kept in mind: these subjects are rather uncommon; the functional status of the nigro-striatal system can be accurately assessed with the use of dopamine transporter imaging with SPECT (decline of the uptake suggests the diagnosis of PD instead of ET), which has become available in clinical practice in many parts of the world (Marshall and Grosset, 2003); these patients can be treated with a combination of the armamentarium used for the two conditions.

## Dystonic Tremor

Until a few years ago there were few allusions to dystonic tremor in the medical literature. In 1998 the Consensus Statement of the Movement Disorder Society on Tremor made a note that “dystonic tremor syndrome is still under debate and different definitions have been proposed” and submitted a description and diagnostic criteria for this entity: (1) tremor in an extremity or body part that is affected by dystonia; (2) focal tremors, usually with irregular amplitudes and variable frequency (mainly less than 7 Hz); (3) mainly postural/kinetic tremors and usually not seen during complete rest (Deuschl et al., 1998).

The issue remained relatively dormant until a number of drug studies enrolling patients with early stages of PD led to the description of the so-called “Scans without evidence of dopaminergic deficit” (SWEDDs; Whone et al., 2003; Holloway et al., 2004; Marek et al., 2005). All these studies, having in common the use of functional imaging of the nigro-striatal system either using PET or SPECT technology and the finding that between 11 and 15% of the patients were SWEDDs. It seems clear now that these patients did not have PD since long-term follow up failed to identify any worsening of the clinical picture and l-DOPA has not improved the tremor (Schneider et al., 2007). SWEDDs comprise a heterogeneous group of patients with a number of underlying causes: dystonic tremor (79%), ET (3%), vascular parkinsonism (3%), and indeterminate in 15% (Bajaj et al., 2012). There are recent reports of genetic defects, such as mutations of GLUT1, presenting with dystonic tremor (Roubergue et al., 2011). More recently, the following features have been reported as characteristic of dystonic tremor: dystonia (which may be subtle), thumb extension tremor, “flurries” or task/position-specificity of tremor, head tremor, dystonic voice, no progression to develop features other than tremor and dystonia, and no clear fatiguing or decrement of repetitive movements (Schneider et al., 2007).

It should be pointed out that the distinction of dystonic tremor from ET is not always simple, with patients with the former being diagnosed with the later (Quinn et al., 2011). This is an area where more work is warranted: one of the problems of the current concept of dystonic tremor is the admission that in some of the patients “tremor is unilateral or very asymmetric, irregular or jerky, position- or task-specific (and therefore more likely to be disabling), relieved by a geste antagoniste, pronation-supination rather than vertical, or occurring in flurries, but without (at least yet) frank dystonia” (Quinn et al., 2011). Despite the fact that the Consensus Statement of the Movement Disorder Society on Tremor (Deuschl et al., 1998) considers isolated head tremor as one feature of ET, clinical practice shows that this phenomenology is almost invariably an expression of dystonia. Obviously, the admission that dystonia may be absent contradicts the current definition of dystonic tremor and, from a practical point of view, renders the differentiation from ET extremely difficult. One should bear in mind that in a few subjects, the clinical picture of dystonia can be dominated almost entirely by tremor with dystonia remaining a very subtle finding. Finally, patients with dystonic tremor often fail to respond to any available pharmacological treatment. It is also important to stress that one potential difficulty in establishing dystonic tremor as a nosological entity on its own is the lack of clinico-pathological studies of this condition. To conclude, it is important to state that in the majority of cases, the distinction between ET and dystonic tremor is readily made. The scenarios discussed herein refer to a limited number of patients.

## Differential Diagnosis of Chorea in Children

Sydenham’s Chorea (SC) accounts for nearly 100% of all cases of chorea in children worldwide (Cardoso et al., 2006; Zomorrodi and Wald, 2006). Typically, the gradual or subacute onset is in the end of the first decade of life, being more frequent in girls than boys and, unlike other manifestations of rheumatic fever, there is a long latency between streptococcal pharyngitis and the development of chorea. Consequently, many patients with SC fail to display laboratory findings of acute infection or inflammatory reaction. The clinical picture comprises motor findings with chorea, and decreased muscle tone. Less common features are tics, present in no more than 8% of subjects, hemichorea (20% of cases), and chorea paralytica (the muscle tone is so decreased that patients are unable to stand) in 8% of our series (Cardoso et al., 2006; de Teixeira et al., 2009). There are also non-motor findings that include obsessions, compulsion, hyperactivity, attention deficit, headache, dysexecutive syndrome and, rarely, psychosis (Maia et al., 2005; Teixeira et al., 2005, 2007; Beato et al., 2010). Of note, disability in SC is usually a result of cardiopathy, present in up to 80% of subjects. Although in the majority of individuals SC comes into remission by the end of the first year of illness, recurrences are seen in up to 20% of patients and persistence of chorea occurs in 25% of subjects (Cardoso et al., 1999).

There has been a steady decline of SC incidence even in areas where it used to be frequent such as Latin America and Africa. This is probably a result of improvement of public health conditions but it may also represent a change in the biology of Streptococcus. The bottom line is that there is a growing need to consider diagnoses alternative to SC in children with chorea. The first differential diagnosis to be considered is vascular chorea. The abrupt onset, lack of behavioral abnormalities and other features suggestive of rheumatic fever and, particularly, abnormal imaging readily distinguish vascular chorea from SC (Cardoso et al., 2006). A particular form of vascular chorea, which is uncommon in the West and more often found in Asia, is moyamoya disease. It is characterized by chronic stenoocclusive vasculopathy of the major vessels of the circle of Willis and development of aberrant distal vascular network (Weinberg et al., 2011). Although the most common clinical features are hemorrhage or ischemia, a few patients may present with paroxysmal chorea. This clinical pattern together with characteristic findings on magnetic resonance imaging and angiography lead to the correct diagnosis (Baik and Lee, 2010). Despite the finding that almost all cases of acute chorea in children are caused by SC, there are instances where alternative diagnoses should be considered. The most important differential diagnosis of SC is chorea associated to systemic lupus erythematosus (SLE) and primary antiphospholipid antibody syndrome (PAPS; Cardoso et al., 2006). In classical forms, patients with SLE have chorea combined with psychosis and extra-neurological features such as rash skin, pericarditis, renal failure, and other organs involvement as well as typical laboratory abnormalities (Reiner et al., 2011; Baizabal-Carvallo and Jankovic, 2012). In PAPS, chorea is associated with headache, behavioral abnormalities, repeated abortion, and thrombotic phenomena such as pulmonary embolism and deep venous thrombosis (Baizabal-Carvallo and Jankovic, 2012). In practice, however, the diagnosis can be challenging since chorea can be the sole finding for a long period of time before the development of other clinical and laboratory findings supportive of the diagnosis of SLE or PAPS (Bakdash et al., 1999); chorea can be a mild feature eclipsed by other findings particularly parkinsonism and myoclonus (Baizabal-Carvallo and Jankovic, 2012); the recurrent nature of SLE and PAPS is also found in at least 1/5 of patients with SC; SC lacks specific laboratory markers. There are also other rare conditions that may mimic SC: vascular disease, prescription and illicit drugs, genetic conditions such as Friedreich’s ataxia and hereditary benign chorea (Cardoso et al., 2006). The practical message is that one needs to be cautious when making the diagnosis of SC, particularly in the absence of other features of rheumatic fever. Patients always require comprehensive laboratory and imaging work up to rule out alternative causes. Finally, clinicians should be attentive to the development of atypical features, even after a long-term follow up, which may suggest SLE, PAPS, or even other causes. Among these features are psychosis, pyramidal signs, ataxia, and ophthalmoparesis.

## Conclusion

In most instances, ET and PD are easily distinguishable. However, there is a small minority of patients with long duration ET who develop parkinsonian features characterized mostly by tremor at rest and rigidity with minor bradykinesia. Current functional imaging, biochemical, and pathological data do not support that these patients have PD. The mechanism underlying parkinsonian features of these patients remains to be determined. Dystonic tremor is an entity growingly diagnosed and regarded as accounting for the majority of SWEDDs in drug trials. Its clinical features, diagnostic criteria and etiology remain controversial and ill-defined. Currently, it is described as the coexistence of tremor and dystonia in a limb or neck. However, as in many reported cases there is coexistent parkinsonian features and very mild dystonia, clinical distinction from PD and even ET may be difficult. Further studies are warranted to improve the accuracy of the diagnosis of dystonic tremor. Finally, although SC is the cause of the majority of acute chorea in children, in some patients SLE or PAPS causes this movement disorder. It is important to keep in mind that not infrequently there is a rather long latency until patients develop clinical and laboratory features that define the diagnosis of these two conditions.

## Conflict of Interest Statement

The author declares that the research was conducted in the absence of any commercial or financial relationships that could be construed as a potential conflict of interest.
